# Frequency and Classification of Femoral Neck Fractures: A Cross-Sectional Study From a Tertiary Care Hospital in Peshawar, Pakistan

**DOI:** 10.7759/cureus.95599

**Published:** 2025-10-28

**Authors:** Muhammad Mannan, Zawar Ahmad, Syed Muneeb Ali Shah, Muhammad Tayyab, Faisal Karim, Rehan Raza Shan, Muhammad Zeeshan Akram

**Affiliations:** 1 Orthopaedic Surgery, Heartlands Hospital, University Hospitals Birmingham NHS Foundation Trust, Birmingham, GBR; 2 Trauma and Orthopaedics, Hayatabad Medical Complex, Peshawar, PAK; 3 Trauma and Orthopaedics, Heartlands Hospital, University Hospitals Birmingham NHS Foundation Trust, Birmingham, GBR; 4 Trauma and Orthopaedics, Bradford Teaching Hospitals NHS Foundation Trust, Bradford, GBR

**Keywords:** avascular necrosis, femoral neck fracture, fracture epidemiology, garden classification, hip fracture, internal fixation, orthopaedic trauma

## Abstract

Introduction: In the human body, the strongest and densest bone is the femur, which plays the pivotal role in weight-bearing. Therefore, femoral neck fractures lead to significant morbidity and mortality worldwide. This study aims to determine the frequency of femoral neck fractures in the general population using the Garden classification.

Methods: This was a descriptive, cross-sectional study conducted at the Department of Orthopaedics, Hayatabad Medical Complex, Peshawar, Pakistan, from January 10 to July 10, 2022. A total of 164 patients, aged 18-70 years of either gender, who presented after trauma with moderate to severe groin pain, tenderness, and swelling, were recruited. Diagnosis was made on the basis of clinical examination and radiological findings. Fractures were then classified using the Garden classification. Data were analyzed using IBM SPSS Statistics for Windows, version 21.0 (IBM Corp., Armonk, New York, United States). Quantitative variables were presented as mean ± SD, and qualitative variables as frequencies and percentages.

Results: The mean age of patients was 50.64 ± 8.12 years. Out of 164 patients, 89 (54.3%) were male and 75 (45.7%) were female. Femoral neck fractures were observed in 51 (31.1%) patients. Among these, Garden Type I fractures were present in 19 (37.3%) patients, Garden Type II fractures in 20 (39.2%), Type III in 8 (15.7%), and Type IV in 4 (7.8%) patients.

Conclusion: The majority of femoral neck fractures in this cohort were classified as Garden Type I and II. Early recognition and classification are essential for guiding treatment and improving outcomes.

## Introduction

The femur is the toughest, longest, and densest tubular bone in the human body, with a prominent feature of load-bearing in the lower extremity. High-energy impacts often cause fractures of the shaft of the femur. Femoral neck fractures are responsible for morbidity and mortality worldwide and are treated surgically. It is crucial to practice effective treatment approaches that meet the important factors, such as age, fracture complexity, and the existence of other injuries [1-3].

Femur neck fractures extensively cause impairment to the ascending cervical arteries of the extracapsular arterial ring [4]. The incidence of femoral fractures is recorded as one among 10,000 annually [5]. On the other hand, its incidence rate increases by three cases per 10,000 population aged below 25 years and above 65 years. In a study by Stockton et al., incidence was more common in male individuals [6].

The common aetiology involves road traffic accidents, gunshots, and falls. Several studies have reported that patients sustaining femoral neck fractures following moderate trauma often have pre-existing reduced bone mineral density or osteopenia, contributing to fracture susceptibility [7]. Various studies reported two types of prominent complications commonly observed after surgical intervention for femoral neck fractures. One is a malunion of the bone, and the second is avascular necrosis (AVN) of the femoral head [8].

The patients affected by AVN complications suffered from heavy economic loss along with severe degradation in the quality of life. The goals of surgical management in these fractures are anatomic reduction and sustainment of fixation. Moreover, it has been observed that the achievement of the treatment depends upon anatomic reduction, fixation stability, and bone [8]. A study was conducted in which neck of femur fracture was observed in 29.71% of patients with road traffic accident as the major cause (62.6%), followed by fall from height (37.3%) [5].

Femur neck fracture involves an important part of the intracapsular femur fracture in the intracapsular part of the proximal femur, which is more frequently observed in the adult population. The focus of the study is to find the frequency of neck of femur fractures in the general population, as not enough literature regarding this topic is available. The outcome of this study will lead to a better understanding and management of this fracture and minimise complications. 

## Materials and methods

This was a prospective descriptive, cross-sectional study conducted in the Department of Orthopaedics at Hayatabad Medical Complex (HMC), Peshawar, Pakistan, to determine the frequency of femoral neck fractures and their different types. The study was carried out over a six-month period, from January 10 to July 10, 2022. It was approved by the Research Evaluation Unit of the College of Physicians and Surgeons Pakistan (approval number: CPSP/REU/OSG-2021-021-2489). Written informed consent was taken from each participant before enrollment. All patients were informed about the purpose, methodology, potential risks, and benefits of the study. Patient's confidentiality and data privacy were strictly maintained throughout the study. For operational consistency, a femoral neck fracture was defined in this study as a fracture line occurring within the hip joint capsule (intracapsular).

Inclusion and exclusion criteria

Patients aged 18-70 years, of either gender, who presented with moderate to severe groin pain, tenderness, and swelling following trauma were included in the study. Diagnosis was made through clinical examination and radiographic evaluation, including anteroposterior and lateral hip X-rays. Patients with extracapsular fractures (intertrochanteric or subtrochanteric), femoral shaft fractures, or periprosthetic fractures were excluded. Moreover, patients with a history of prior surgical intervention on the affected femur and those who refused to participate or did not provide written informed consent were also excluded. The eligibility criteria are given in Table 1.

**Table 1:** Inclusion and exclusion criteria

Inclusion Criteria	Exclusion Criteria
Patients aged 18 to 70 years	Patients with extracapsular fractures (intertrochanteric, subtrochanteric)
Both male and female patients	Femoral shaft fractures
Patients presenting with moderate to severe groin pain, tenderness, and swelling following trauma	Periprosthetic fractures
Femoral neck fracture defined as fracture line occurring within the hip joint capsule (intracapsular), confirmed by clinical examination and radiographs	Patients with previous surgical intervention on the affected femur
-	Patients who declined or failed to provide written informed consent

Sampling technique and sample size calculation

Patients were selected by a non-probability consecutive sampling technique. All the patients fulfilling the inclusion criteria who presented during the study period were included in the study until the required sample size was achieved. Patients with incomplete clinical or radiographic data were excluded to ensure accuracy and consistency of classification.

WHO sample size determination software was used to calculate the sample size [9]. A total of 164 patients were recruited. This calculation was based on an expected prevalence of femoral neck fractures of 29.71% [5], with a 95% confidence level and a 7% margin of error.

Data collection

A detailed clinical assessment and radiological evaluation were done for each participant, including anteroposterior and lateral hip X-rays. Femoral neck fractures were diagnosed according to the operational definition, and fracture types were classified using the Garden classification system. Data recorded included age, gender, and the presence of comorbid conditions such as diabetes mellitus, hypertension, and cardiovascular disease. These variables were documented on a standardized data collection proforma. All patients received appropriate treatment as per institutional protocols and clinical guidelines.

Statistical analysis

Data analysis was conducted using IBM SPSS Statistics for Windows, version 21.0 (IBM Corp., Armonk, New York, United States). Quantitative variables such as age were presented as mean ± standard deviation (SD). Categorical variables, including gender, presence of femoral neck fracture, fracture classification, and comorbidities, were summarized as frequencies and percentages.

Stratification was performed to evaluate potential associations between femoral neck fractures and demographic or clinical variables such as age group, gender, and comorbidities. The Chi-square test or Fisher’s exact test was used as appropriate. A p-value < 0.05 was considered statistically significant.

## Results

In our cohort, using 50 years as the age threshold allowed us to separate younger, predominantly high-energy trauma cases from older patients with more fragility-related injuries. There were 89 male patients (54.3%) and 75 female patients (45.7%). Regarding comorbidities, 25 patients (15.2%) had diabetes mellitus, 22 (13.4%) were hypertensive, and 27 (16.5%) had cardiovascular disease. Femoral neck fractures were diagnosed in 51 patients (31.1%), while 113 patients (68.9%) did not have this fracture type. This is presented in Table 2.

**Table 2:** Demographic and clinical profile of patients (N=164)

Variable	Category	Frequency (Percentage)
Age (years)	18–50 years	67 (40.85%)
	51–70 years	97 (59.15%)
	Mean ± SD	50.640 ± 8.12
Gender	Male	89 (54.3%)
	Female	75 (45.7%)
Diabetics	Yes	25 (15.2%)
	No	139 (84.8%)
Hypertensive	Yes	22 (13.4%)
	No	142 (86.6%)
Cardiovascular Diseases	Present	27 (16.5%)
	Absent	137 (83.5%)
Neck of Femur Fracture	Yes	51 (31.1%)
	No	113 (68.9%)

Among the patients aged 18-50 years, 15 (22.4%) had femoral neck fractures, compared to 36 (37.1%) in the 51-70 years group, indicating a statistically significant association with increasing age (p = 0.045). Although a higher proportion of males (32 out of 89; 36%) had femoral neck fractures than females (19 out of 75; 25.3%), this difference was not statistically significant (p = 0.143). The presence of comorbidities showed a strong correlation with fracture incidence. Among diabetic patients, 17 out of 25 (68%) had femoral neck fractures compared to 34 out of 139 (24.5%) nondiabetic patients (p < 0.001). Similarly, 13 out of 22 (59.1%) hypertensive patients sustained fractures versus 38 out of 142 (26.8%) non-hypertensives (p = 0.002). Cardiovascular disease was also significantly associated with fracture occurrence; 17 out of 27 (63%) patients with cardiovascular disease had femoral neck fractures compared to 34 out of 137 (24.8%) without (p = 0.002). This is presented in Table 3.

**Table 3:** Stratification of type of neck of femur fracture by patient characteristics The p-values were calculated using the Chi-square test; where assumptions were not met, the Fisher’s exact test was applied. A p-value < 0.05 was considered statistically significant.

Variable	Category	Neck of Femur Fracture, n (%)	No Neck of Femur Fracture, n (%)	p-value
Age	18–50 years	15 (22.4%)	52 (77.6%)	0.045
	51–70 years	36 (37.1%)	61 (62.9%)	
Gender	Male	32 (36%)	57 (64%)	0.143
	Female	19 (25.3%)	56 (74.7%)	
Diabetes	Diabetic	17 (68%)	8 (32%)	0.000
	Non-Diabetic	34 (24.5%)	105 (75.5%)	
Hypertension	Hypertensive	13 (59.1%)	9 (40.9%)	0.002
	Non-Hypertensive	38 (26.8%)	104 (73.2%)	
Cardiovascular disease	Present	17 (63%)	10 (37%)	0.002
	Absent	34 (24.8%)	103 (75.2%)	

Among the 51 patients diagnosed with femoral neck fractures, the majority were classified as Garden Type I and Type II. Specifically, 19 patients (37.3%) had Type I fractures, while 20 patients (39.2%) had Type II fractures. Type III fractures were observed in eight patients (15.7%), and Type IV fractures in four patients (7.58%). The distribution of fracture types did not show a statistically significant difference (p = 1.000), indicating that the prevalence of each Garden classification type occurred within expected variation among the study population. This is presented in Table 4.

**Table 4:** Stratification of neck of femur fracture with respect to fracture type (N=51) The p-value was calculated using the Chi-square test to assess the distribution of fracture types. A p-value < 0.05 was considered statistically significant.

Type of Neck of Femur Fracture	Frequency	Percentage	p-value
I	19	37.3%	1.000
II	20	39.2%	
III	8	15.7%	
IV	4	7.58%	

## Discussion

Femur neck fractures were initially classified by Sir Astley Cooper in 1823 into two categories: intra-capsular and extra-capsular, with prognostic implications [10]. Another form of classification, the Pauwels classification, was first proposed in 1935 [11]. It is a biomechanical system dividing femoral neck fractures on the basis of the inclination of the fracture line relative to the horizontal. It includes Type I (< 30°), Type II (30-50°), and Type III (> 50°). Bartoníček (2001) gave the definitive interpretation and angle definitions for each category [12]. With increasing angle, forces acting on the fracture line shift from compressive to shearing, which increases the risks of displacement, non-union after reduction, and fixation failure [11].

A British orthopaedic surgeon, Robert Symon Garden, in 1961, introduced a more comprehensive classification system, based on the degree of displacement, the completeness of the fracture, and the alignment of the trabeculae in the head and neck of the femur [13]. Garden’s research, which analysed 80 patients, categorized femoral neck fractures into four types (I-IV). He tracked the outcomes for at least 12 months post-surgery. Fractures classified as class I and II achieved 100% union rates, while class III and IV had union rates of 92% and 58%, respectively. On the basis of anteroposterior (AP) radiographs of the hip, Garden's classification distinguishes fractures as incomplete and valgus impacted, complete and nondisplaced, complete and partially displaced, and complete and fully displaced [14].

Over time, Garden’s classification was simplified by clinicians, who began to categorize femoral neck fractures as either displaced or nondisplaced [15,16]. Level of displacement would typically guide treatment decisions [17,18]. For nondisplaced fractures (Types I and II), internal fixation is typically recommended to maintain the integrity of the femoral head. Bentley found that nondisplaced impacted fractures can displace in up to 15% of cases [19]. So he recommended internal fixation for Garden Types I and II fractures for achieving early weightbearing and avoiding avascular necrosis. Fixation methods include cannulated screws, dynamic hip screws, proximal femoral locking plates, and cephalo-medullary nails [20,21]. A randomised multicentre trial, the Fixation using Alternative Implants for the Treatment of Hip fractures (FAITH) study, compared sliding hip screws and cancellous screws for the treatment of femoral neck fractures [22]. The study revealed a greater incidence of avascular necrosis in the sliding hip screw group (9%) compared to the cancellous screw group (5%); however, there was no statistically significant difference in the overall re-operation rates between the two groups.

For displaced femoral neck fractures (Garden Types III and IV), treatment options include hemiarthroplasty, total hip arthroplasty (THA), or internal fixation. In elderly patients, arthroplasty is preferred, while younger patients typically benefit from internal fixation. A meta-analysis by Lu-Yao et al. highlighted a higher rate of nonunion (33%) and avascular necrosis (16%) following primary internal fixation for displaced fractures [23]. In a study by Tidermark et al., comparing internal fixation and THA in old age patients with displaced femoral neck fractures, revision rates were significantly higher in the internal fixation group (42.5%) compared to the arthroplasty group (5%) [24]. Current trends favour arthroplasty, either hemiarthroplasty or THA, for displaced femoral neck fractures in elderly patients. In the multicentre randomised trial by Keating et al., there were no significant differences in mortality between fixation, bipolar hemiarthroplasty, and THA throughout the two-year follow-up period [25]. For patients over 60, not actively mobile, with comorbidities, hemiarthroplasty can provide satisfactory functional outcomes. Meanwhile, more active patients may benefit from THA due to its better functional outcomes and lower percentage of re-operation [7]. For individuals under 60, femoral head preservation through internal fixation is considered more appropriate due to the higher functional demands [26].

Van Embden et al. demonstrated that simplifying Garden's four-type system into two categories-non-displaced (Type I-II) vs. displaced (Type III-IV) increased multi-rater reliability (κ from 0.31 to 0.52), indicating better inter-observer agreement [15]. A survey by Zlowodzki et al. suggested simplifying the Garden system into just two categories: undisplaced (Type I-II) and displaced (Type III-IV), which has been shown to improve inter-observer agreement, with 96% of surveyed surgeons able to reliably distinguish between these broader groups compared to only 39% who could differentiate all four types [16]. In another study, interobserver agreement on the Garden classification was only 22%, but there was 77% agreement when fractures were categorised as simply displaced or nondisplaced [21]. Other classification systems, such as Pauwels and AO (Arbeitsgemeinschaft für Osteosynthesefragen), have also been used, with variable reliability. Turgut et al. (2016) observed even lesser inter-observer agreement for Pauwels classification (κ = 0.24 and 0.18), thus limiting reproducibility. In studies comparing the Garden classification with the Pauwels and AO systems, intra-observer reliability was found to be highest for the Garden classification, with a Kappa value of 0.759 [27].

Femoral neck fractures that occur in the setting of polytrauma are beyond classification and determination of frequency because associated injuries can complicate diagnosis and management. Recent literature has highlighted the prognostic significance of complex trauma patterns such as floating knee injuries and pelvic trauma, underscoring the need for comprehensive evaluation in these patients [28-31]. Furthermore, outcomes after proximal femoral fixation, including postoperative pain, union rates, and implant stability, have been studied in greater detail, and the findings indicate that both implant choice and fracture stability can significantly influence recovery [32,33]. Although the current study did not assess outcomes or management, the observed associations between femoral neck fractures and comorbidities such as diabetes, hypertension, and cardiovascular disease highlight an important area for future research

Limitations

Despite providing valuable insights into the frequency and classification of femoral neck fractures, this study has certain limitations. First, the study was carried out at a single tertiary care centre, which may limit the generalisability of the findings to other settings, particularly rural or primary care facilities. Second, the use of non-probability consecutive sampling introduces a risk of selection bias, which may have influenced the observed distribution of fracture types and associated risk factors. Third, although the Garden classification system was used due to its extensive clinical use, it is subject to interobserver variability, which may affect the consistency of fracture categorisation. The study did not use multiple reviewers or kappa statistics to assess inter-rater reliability. Additionally, radiological assessment was limited to plain anteroposterior and lateral X-rays, and advanced imaging techniques, such as CT or MRI, were not used, which could have provided more detailed fracture characterisation. Another limitation of our study is that it focused solely on the frequency and classification of femoral neck fractures without assessing management strategies or patient outcomes. This restricted scope prevents us from drawing correlations between fracture type, treatment modality, and prognosis.

## Conclusions

This study concludes that femoral neck fractures were most frequently classified as Garden’s Type I and II in our cohort. By documenting the frequency and distribution of fracture types, the study highlights the continued relevance of the Garden classification system in clinical practice. While this classification remains widely used by orthopedic surgeons, our findings emphasize the need for further research that incorporates management strategies and long-term outcomes.
